# Psychological Interventions for Post-traumatic Stress Symptoms in Psychosis: A Systematic Review of Outcomes

**DOI:** 10.3389/fpsyg.2017.00341

**Published:** 2017-03-14

**Authors:** Sarah Swan, Nadine Keen, Nicola Reynolds, Juliana Onwumere

**Affiliations:** ^1^South London and Maudsley NHS Foundation TrustLondon, UK; ^2^Department of Psychology, Institute of Psychiatry, Psychology and Neuroscience, King's College LondonLondon, UK

**Keywords:** psychosis, SMI, trauma, post-traumatic stress disorder, PTSD, therapy, psychological intervention

## Abstract

Individuals with severe mental health problems, such as psychosis, are consistently shown to have experienced high levels of past traumatic events. They are also at an increased risk of further traumatisation through victimization events such as crime and assault. The experience of psychosis itself and psychiatric hospitalization have also been recognized to be sufficiently traumatic to lead to the development of post-traumatic stress (PTS) symptoms. Rates of post-traumatic stress disorder (PTSD) are elevated in people with psychosis compared to the general population. The current guidance for the treatment of PTSD is informed by an evidence base predominately limited to populations without co-morbid psychiatric disorders. The systematic review therefore sought to present the current available literature on the use of psychological treatments targeting PTS symptoms in a population with a primary diagnosis of a psychotic disorder. The review aimed to investigate the effect of these interventions on PTS symptoms and also the effect on secondary domains such as psychotic symptoms, affect and functioning. Fifteen studies were identified reporting on cognitive behavior therapy, prolonged exposure, eye movement desensitisation and reprocessing and written emotional disclosure. The review provides preliminary support for the safe use of trauma-focused psychological interventions in groups of people with severe mental health problems. Overall, the interventions were found to be effective in reducing PTS symptoms. Results were mixed with regard to secondary effects on additional domains. Further research including studies employing sufficiently powered methodologically rigorous designs is indicated.

## Introduction

For the majority of individuals in the general adult population, one traumatic experience is likely to occur within their lifetime (Frans et al., 2005; Breslau, 2009). The associated distress is mostly short-lived and diminishes of its own accord (Bisson, 2007). For a proportion, however, distress can continue and symptoms of post-traumatic stress (PTS) can develop. A PTS response is characterised by a number of core symptoms including: intrusive or re-experiencing symptoms such as flashbacks and nightmares; persistent cognitive and/or behavioural avoidance; negative changes to cognition and affect, and a marked increase in arousal and reactivity such as hypervigiliance and exaggerated startle (American Psychiatric Association, 2013). Post-traumatic stress disorder (PTSD) is diagnosed when a person presents with a combination of symptoms from these core symptom groups, typically within the context of increased distress and disturbance to functioning. Approximately 3% of the general adult population is estimated to have PTSD and an additional 3.6% are thought to experience PTS symptoms which do not meet full diagnostic criteria for a diagnosis of PTSD i.e., sub-threshold (McLaughlin et al., 2015). The diagnosis is associated with significant disturbance to occupational and social functioning (Karam et al., 2014), increased substance misuse (Bisson, 2007), higher suicidality (Sareen et al., 2007) and increased health and social service use (Atwoli et al., 2015).

PTSD is highly co-morbid with other psychiatric diagnoses (Greene et al., 2016) and the co-occurrence of PTS symptoms and more severe mental health difficulties is an area of increasing interest. Individuals with psychosis, in particular, are consistently shown to have experienced high levels of trauma (Lommen and Restifo, 2009; Varese et al., 2012). This group also have an increased risk of continued exposure to traumatic events. Rates of victimisation have been reported to be between 2.3 and 140.4 times higher in people with severe mental illness (SMI) than in the general population; with this vulnerability thought to arise from their current mental state and associated social context such as poverty, homelessness, and social isolation (Maniglio, 2009). There has also been an increasing recognition of the traumatising effects of psychiatric hospitalisation and psychotic symptoms. Significant numbers of individuals with psychosis are shown to develop a PTS response to symptoms of psychosis or hospital experiences severe enough to meet diagnostic criteria for PTSD (Berry et al., 2013). Experiences such as being given medication against one's will, being detained under the Mental Health Act (1983, as amended in 2007) (Tarrier et al., 2007) and threatening auditory hallucinations (Beattie et al., 2009) can lead to PTS symptoms. Following the latest changes to PTSD diagnostic criteria in DSM-V (American Psychiatric Association, 2013), in which the stressor criterion A2: “*the person's response involved intense fear, helplessness or horror*” was removed, controversy remains over whether hospitalisation or symptom related experiences can be sufficiently categorised within the trauma criterion (Jackson et al., 2004). However, the debate regarding DSM changes to criterion A remains outside the scope of this review; for further discussion see published works e.g., Friedman et al. (2011) and Karam et al. (2010). Regardless of this debate, given the high exposure to traumatic events that people with severe mental health problems typically have endured, it is unsurprising that the prevalence of PTS symptoms in this group is higher than that of the general population (Mueser et al., 2002).

The finding that a substantial proportion of people with psychosis and other severe mental health problems can present with PTS symptoms is of growing importance in clinical settings. In the recently updated treatment guidance on psychosis and schizophrenia from the National Institute for Health and Care Excellence (NICE) (National Institute for Health and Care Excellence, 2014), it is acknowledged that individuals are likely to have experienced trauma through events related to the development of psychosis and/or trauma as a direct result of the psychosis itself. Consequently the guidance calls for all service users to be routinely assessed for PTS symptoms. Screening for secondary co-morbid mental health difficulties is of particular importance due to the associated poorer outcome for individuals with multiple mental health difficulties (Buckley et al., 2009). For individuals with psychosis and co-morbid PTSD specifically, there is a positive correlation with increased cognitive, affective and behaviour disturbance (Seedat et al., 2003), reduced quality of life and greater acute service use (Grubaugh et al., 2011). There are also the implications of cost related directly to service use and the wider economic burden incurred through loss of ability to work and welfare (Insel, 2008).

The NICE clinical guidance for PTSD management (National Institute for Health and Care Excellence, 2005) recommends the use of trauma-focused cognitive behaviour therapy (TF-CBT) and TF-CBT and/or eye movement desensitisation and reprocessing (EMDR) as the first line treatment for PTS symptoms present for less than 3 months and those present for longer periods, respectively. The evidence base for the efficacy of TF-CBT and EMDR in reducing PTS symptoms is well established within an adult population (Bisson and Andrew, 2005). As with many intervention outcome trials, strict inclusion criteria are often employed to achieve a homogenous group in an attempt to reduce variance or confounding factors that may arise in an increasingly heterogeneous sample (Green, 2006). In practice, trials tend to include participants with a sole diagnosis of PTSD, with co-morbidity an exclusion criterion. Diagnosis of a psychotic condition is the most common exclusion criterion within many randomised controlled clinical trials (de Bont et al., 2013a). Though the co-occurrence of PTS symptoms and psychosis is emerging as a relatively common phenomenon, the evidence base is mostly limited to individuals without co-morbid conditions. Where the evidence base and clinical guidance is orientated to single morbidity, practitioners are placed in a position where they must rely upon their clinical judgement in treatment decision making (Hughes et al., 2013). It thus follows that mental health professionals are arguably faced with the difficulty of having to infer whether the generally recommended trauma treatments are appropriate for individuals with psychosis. This is further complicated by the fact that therapists are often reluctant to treat PTS symptoms due to concerns that the experiential reprocessing of trauma may exacerbate psychotic symptoms (Becker et al., 2004; Gairns et al., 2015).

The review therefore seeks to complement the existing trauma intervention literature (e.g., Bisson and Andrew, 2005; Mabey and Servellen, 2014; Sin and Spain, 2016) by presenting the breadth of current evidence for the use of psychological interventions targeting PTS symptoms within people with a primary diagnosis of psychosis. The systematic review will seek to address the following questions: (i) what are the psychological interventions with published data on their use to treat PTS symptoms in people with psychosis; (ii) how effective are these treatments in treating PTS symptoms; and (iii) how effective are these treatments in bringing about change in co-morbid psychiatric symptoms or secondary domains (i.e., psychosis, depression, anxiety, functioning).

## Methods

### Criteria and definitions

#### Study inclusion

Studies were selected for consideration in the review if they presented outcome data on the effect of one or more psychological interventions targeting PTS symptoms in adults with psychosis. Studies employing a randomised controlled trial design, non-randomised controlled, un-controlled, case series and single n methodology were included. Studies were required to be published in peer reviewed journals with the abstract and content written in English. Studies which did not report on a measure of PTS symptoms were excluded.

#### Population

Studies for inclusion were required to present data on individuals over the age of 16 years old with a primary diagnosis of a psychotic disorder (including schizophrenia and related disorders, schizoaffective disorder, non-organic psychoses according to either DSM or ICD criteria). Studies were not restricted to those reporting on homogenous psychosis populations. Studies could be included if they reported on a mixed SMI group as long as they included individuals with psychosis. Such studies were included due to the limited evidence base and the authors' intention to provide a representative reflection of the emerging evidence. Studies were not excluded if the population had additional co-morbid disorders, such as depression, anxiety or Axis II diagnoses.

Studies were required to include a population as outlined above with: (i) an additional co-morbid diagnosis of PTSD, according to DSM or ICD criteria; or (ii) the presence of PTS symptoms as indicated by standardised measures. The studies for consideration were not limited on the basis of PTS symptom severity, duration or nature of the traumatic event. Regarding the latter, the index traumatic event could include the experience of symptoms of psychosis or events relating to hospitalisation in addition to more “traditional” notions of a traumatic event as defined by diagnostic criteria.

#### Psychological intervention

Psychological interventions were defined for this review as any non-pharmacological treatment specifically aiming to target psychological processes implicated in contributing to symptoms of psychological distress. Treatments were required to be based on psychological theories or models of psychopathology and/or include a clearly defined protocol of treatment (including behavioural, cognitive or other psychotherapeutic techniques) or a theoretical hypothesis for treatment efficacy. An intervention could be delivered within an inpatient or community setting, individually or in a group and be therapist or non-therapist led.

### Search

#### Procedure

The search methods employed to identify potentially relevant studies according to the inclusion criteria and definitions involved the computerised searching of four widely used electronic databases: Embase, PsychINFO, MEDLINE, and Web of Science. Advanced keyword search strategy was conducted by combining the following terms: (“psychosis” OR “psychotic disorder” OR “schizophren^*^”) AND (“ptsd” OR “trauma^*^” OR “post trauma^*^” OR “post-trauma^*^” OR “life event” OR “acute stress”) AND (“counsel^*^” OR “psychological therapy” OR “psychotherapy” OR “talking therapy” OR “intervention”). Regarding the PTS search, a broad selection of terms were included to reflect the heterogeneous use of terminology within the literature, where a number of different descriptors are often employed to describe the same phenomenon.

Additional identification of potential studies was conducted through manual screening of relevant reviews, personal communication with study authors and searching of reference lists of the articles selected for inclusion in the review.

#### Screening methodology

Records generated from the electronic search were exported to bibliographic software and duplicates removed electronically. A second screening was conducted manually to remove remaining duplicate records. Articles were chosen for inclusion by firstly screening the titles, and then by reviewing abstracts of titles deemed potentially relevant. Full text articles were then sourced for potentially relevant records and read in full to determine if the study met the full review inclusion criteria. All stages of screening were undertaken independently by the authors. In cases where there was a disagreement, discussion between the authors took place until consensus was reached.

### Quality assessment

Study quality was assessed using the Clinical Trial Assessment Measure (CTAM) (Tarrier and Wykes, 2004). This quality assessment tool is designed to consider the key methodological and design factors within psychotherapeutic intervention trials in mental health research. The assessment tool includes 15 items, falling under six methodological categories: sample size/method of recruitment, treatment allocation, assessment of outcome, control groups, treatment description and analysis. The measure has been used within reviews of psychological interventions (Gregg and Tarrier, 2007; Tarrier et al., 2008) and in reviews specifically within psychosis (Tarrier and Wykes, 2004; Wykes et al., 2008, 2011).

## Results

### Search results

**Figure 1:**
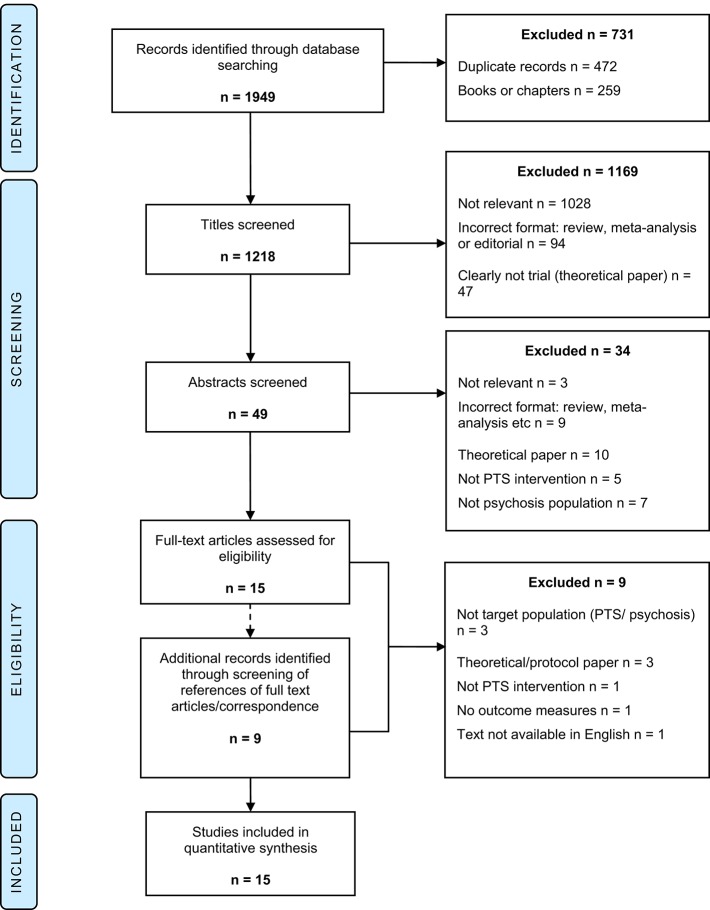
**PRISMA flow diagram**.

A total of 1,949 studies were identified from electronic database searching. Following the removal of duplicate records, 1,477 remained. Records that were not journal articles (i.e., books or book chapters) were then excluded resulting in a pool of 1,218 records which were then screened for eligibility. Full text screening was conducted for 15 articles. At this stage the articles' references were reviewed to identify additional relevant records. Via this method and through direct correspondence with article authors, an additional nine records were identified and full text screened. Fifteen studies were selected to be included in the final review (see Figure 1 for PRISMA flow diagram) (Moher et al., 2009).

### Overview of included studies

The studies included in the review varied in study design. Two articles employed single n methodology reporting on a single case study and two studies reported on a case series of two and three participants, respectively. The remaining articles included five un-controlled studies, one non-randomised controlled study and five randomised controlled trials (RCT).

Supplementary Table 1 provides a summary of the characteristics and main findings of each article in the review. Seven studies were conducted in the United States of America (USA), four in the United Kingdom, three in the Netherlands and one in Australia. Eleven studies investigated CBT or a CBT informed intervention e.g., cognitive recovery intervention (CRI), two PE and EMDR, one EMDR alone, and one the use of written emotional disclosure. Twelve studies reported on interventions delivered individually, two via a group format and one study used a mixed group and individual therapy implementation.

### Sample characteristics

The studies reported on a total sample of 585 participants; with the total number of participants per study ranging from 1 to 155. The age of the seven participants in the case studies/series ranged from 31 to 56 years. Of the trial papers, the mean age was 39.7 years (SD 8.5); data were available for 10 of the 11 articles. Fourteen of the studies reported on demographic gender information (*n* = 537); with 59.6% of the sample being female. Eight studies reported on participant ethnicity; where the percentage of non-white participants per study ranged from 0 to 58%. Twelve studies recruited participants from community mental health services, two from inpatient services and one from both community and inpatient services.

Four studies (case studies and case series) did not employ inclusion or exclusion criteria by virtue of the research design. The case studies reported on individuals with a primary diagnosis of paranoid schizophrenia (Kayrouz and Vrklevski, 2015) and schizophrenia (Kevan et al., 2007); where the presence of significant PTS symptoms was confirmed using self report measures. The case series reported on two participants both meeting ICD-10 criteria for schizophrenia and PTSD (Callcott et al., 2004) and on three individuals all meeting DSM-IV criteria for PTSD; one with a primary diagnosis of bipolar affective disorder (BPAD) and two with a diagnosis of schizoaffective disorder (Hamblen et al., 2004). The remaining 11 studies varied in their inclusion criteria regarding primary diagnostic categories (see Table 1). The number of participants with a primary diagnosis of a psychotic disorder, as opposed to another diagnosis meeting criteria for SMI such as a mood disorder, ranged from 15.7 to 100% of the sample. Two studies reported solely on participants experiencing a first episode of psychosis (Bernard et al., 2006; Jackson et al., 2009).

**Table 1:** **Primary diagnosis inclusion criteria of included studies**.

**Study**	**Primary diagnosis inclusion criteria**
Callcott et al., 2004; Hamblen et al., 2004; Kevan et al., 2007; Kayrouz and Vrklevski, 2015	Not applicable as case study/case series
Rosenberg et al., 2004	DSM-IV diagnosis of schizophrenia, schizoaffective disorder, major depression or psychotic disorder not otherwise specified
Bernard et al., 2006; Jackson et al., 2009	ICD-10 diagnosis of schizophrenia, persistent delusional disorders, acute and transient psychotic disorders or schizoaffective disorders (ICD-10 F20, F22, F23, F25)
Mueser et al., 2007	Severe mental illness as defined by DSM-IV Axis I or II disorder and functional impairment with respect to ability to work or care for oneself
Trappler and Newville, 2007; Frueh et al., 2009	DSM-IV diagnosis of schizophrenia or schizoaffective disorder
Mueser et al., 2008; Lu et al., 2009	DSM-IV diagnosis of schizophrenia, schizoaffective disorder, major depression or bipolar disorder
van den Berg and van der Gaag, 2012	Schizophrenia Spectrum Disorder (Diagnostic system not specified)
de Bont et al., 2013a; van den Berg et al., 2015	DSM-IV diagnosis of a psychotic disorder or mood disorder with psychotic features

Eight of the 11 studies required participants to have a secondary formal diagnosis of PTSD (Rosenberg et al., 2004; Mueser et al., 2007, 2008; Frueh et al., 2009; Lu et al., 2009; de Bont et al., 2013a; van den Berg et al., 2015). In one study it was reported that participants had co-morbid PTSD but it was unclear as to whether this was formally diagnosed or indicated through self-report measures (Trappler and Newville, 2007). Two studies did not stipulate the necessity of a baseline significant presence of PTS symptoms; however trauma-related symptoms were measured and identified as the target of the interventions (Bernard et al., 2006; Jackson et al., 2009).

### Description of interventions

#### Cognitive behaviour therapy (CBT)

CBT interventions targeting PTS symptoms, as described in the literature, have in common four key elements: psycho-education, anxiety management; exposure and cognitive restructuring. The way in which any one of these components is delivered or the extent to which they are emphasised within the intervention varies from one study to another. Four studies in the review described the heterogeneous implementation of a CBT intervention; one study focused on cognitive restructuring following initial written elaboration of trauma memory (Kevan et al., 2007), one study incorporated the use of Smucker's child sexual abuse imagery rescripting (Callcott et al., 2004), one study predominately used exposure preceded by Cloitre's Skill Training in Affect Regulation preparatory work (Trappler and Newville, 2007) and the final study from Kayrouz and Vrklevski (2015) drew on schema therapy ideas.

Six studies reported on the use of a CBT protocol developed specifically for the treatment of PTS symptoms in individuals with SMI (Hamblen et al., 2004; Rosenberg et al., 2004; Mueser et al., 2007, 2008; Frueh et al., 2009; Lu et al., 2009). The main components of the protocol designed by Frueh et al. (2009) were psycho-education, anxiety management, social skills training and exposure therapy. The article described the intervention delivered in a mixed group and individual format over an 11 week period. The exposure element was delivered individually, with the preceding therapy components initially delivered in a group. Four studies used the SMI protocol outlined by Mueser et al. (Lu et al., 2009) which was delivered individually. The protocol summarised an eight stage modular intervention, delivered over 12–16 sessions, broadly grouped into five parts: introduction/engagement, breathing retraining, psycho-education, cognitive restructuring and termination. One study (Mueser et al., 2007) used an adapted version of this protocol delivered in a group over an increased number of sessions.

One of the 11 studies (Jackson et al., 2009) reported on cognitive recovery intervention (CRI); a CBT informed intervention. CRI is a modular protocol based therapy designed to aid psychological recovery and adjustment to first episode psychosis over a period of 6 months (with a maximum of 26 sessions). The three main elements of CRI include engagement/formulation, trauma processing and psychotic illness appraisal (shame, loss, entrapment). Appraisals relating to shame, loss and entrapment have been implicated in the development and maintenance of PTS symptoms (Lu et al., 2009). Hence, cognitive techniques such as developing alternative beliefs are used to challenge and bring about change in these appraisals.

#### Prolonged exposure (PE)

Two studies reported on PE (de Bont et al., 2013a; van den Berg et al., 2015). PE is an approach which involves the systematic exposure to previously avoided trauma related stimuli, either via imaginal exposure or *in-vivo* means. Both studies reported on PE delivered in accordance with the manual by Foa et al. (2007), in 90 min sessions. Therapy sessions involved initial case conceptualisation, development of an exposure hierarchy and then repeated exposure within the remaining sessions. In addition, exposure was continued outside of session by listening to audio recordings of the exposure 5 days a week.

#### Eye movement desensitisation and reprocessing (EMDR)

EMDR was evaluated in three studies (van den Berg and van der Gaag, 2012; de Bont et al., 2013a; van den Berg et al., 2015). All employed the use of the Dutch translation of the standard eight phase EMDR protocol (Shapiro, 2001). Broadly this protocol involves the following: history and treatment planning; preparation of self-control techniques and engagement; assessment and identification of trauma memories; desensitisation including bilateral stimulation typically via visual tracking of the therapist moving their fingers back and forth; installation of positive cognition; body scan; closure involving the implementation of self-control techniques; re-evaluation and review (Menon and Jayan, 2010).

#### Written emotional disclosure

One study (Bernard et al., 2006) reported on the use of written emotional disclosure; where individuals are invited to provide a written account of traumatic experiences. The study used an adapted protocol from Pennebaker and Beall (1986) where participants were asked to write specifically about the experience of psychosis and the related treatment that was perceived as the most stressful and upsetting, doing so for 15 min at three separate time points. The intervention is entirely led by the individual and is not delivered by a therapist.

### Outcomes

#### PTS symptoms and trauma related measures

For the purpose of this review, measures and results have been categorised into PTSD/ PTS symptoms and measures of all other domains. It should be noted that this is not necessarily the categorisation used within the original articles (for example some studies have included measures of mood as a primary outcome) however to enable clarity in presenting the findings, this crude grouping has been applied and the implications reviewed in the discussion section.

In all 15 studies, the primary outcome was PTSD diagnosis or PTS symptoms. In seven studies (46.7%), where the outcome measure was clinician rated, the Clinician Administered PTSD Scale (CAPS) was used. The remaining eight studies (53.3%) used self-report measures of PTS symptoms (see Supplementary Table 1 for measures used).

#### Secondary domains

Eight studies (53.3%) reported on general psychopathology and distress (Callcott et al., 2004; Hamblen et al., 2004; Rosenberg et al., 2004; Trappler and Newville, 2007; Mueser et al., 2008; Frueh et al., 2009; Lu et al., 2009; de Bont et al., 2013a). Ten studies (66.7%) (Callcott et al., 2004; Bernard et al., 2006; Kevan et al., 2007; Mueser et al., 2007, 2008; Frueh et al., 2009; Jackson et al., 2009; Lu et al., 2009; van den Berg and van der Gaag, 2012; Kayrouz and Vrklevski, 2015) reported on symptoms of depression. Four studies included measures of anxiety. Three studies included measures of psychotic symptoms (Callcott et al., 2004; van den Berg and van der Gaag, 2012; de Bont et al., 2013b). Additional secondary outcomes such as recovery style and insight (Bernard et al., 2006), working alliance (Mueser et al., 2008), anger and satisfaction (Frueh et al., 2009), self-esteem (Jackson et al., 2009; van den Berg and van der Gaag, 2012) and social functioning (de Bont et al., 2013a) were included.

### Quality assessment

The outcome of the quality assessment for each study included in the review is presented in Table 2. The CTAM total scores ranged from 9 to 91 (with a maximum score of 100). The mean total CTAM score across all studies was 39.67 (SD 28.38). The methodology employed across the studies varied greatly.

**Table 2:** **Quality assessment CTAM ratings for papers categorized by study design**.

**Design**	**Study**	**Total CTAM score**	**Mean Score (SD)**
Case study Case series	Callcott et al., 2004	9	11.00 (2.45)
	Hamblen et al., 2004	12	
	Kevan et al., 2007	14	
	Kayrouz and Vrklevski, 2015	9	
Un-controlled study	Rosenberg et al., 2004	28	29.80 (4.27)
	Mueser et al., 2007	34	
	Frueh et al., 2009	34	
	Lu et al., 2009	24	
	van den Berg and van der Gaag, 2012	29	
Controlled study (non-randomised)	Trappler and Newville, 2007	26	n/a
Controlled study (RCT)	Bernard et al., 2006	67	75.20 (15.07)
	Mueser et al., 2008	87	
	Jackson et al., 2009	77	
	de Bont et al., 2013a	54	
	van den Berg et al., 2015	91	
	Grand mean (SD)	39.67 (28.38)	

The case studies and case series all tended to employ standardised measures and reported on a protocol or described the intervention used in detail. Case studies and case series did not include blinded assessment of outcome and most did not employ statistical methods to evaluate the outcome of the intervention therefore were mostly judged not to have employed appropriate analyses strategies. The five un-controlled studies failed to report on power analyses and had small sample sizes excluding one study. By nature of their design these studies did not include randomisation and were all deemed to use appropriate methods of analysis however the adequate management of drop outs varied. All studies provided a description of the intervention however some studies did not report on adherence to protocol. Of the six controlled studies, half reported on power analyses or included more than 27 participants in each group and all but one involved random group allocation with most describing this process. Of the five RCTs all but one used standardised measures conducted by a blinded independent assessor. There was greater consistency in these studies to use adequate analysis strategies and manage participants that had dropped out in the analysis. All RCTs used and described a protocol driven intervention and reported on therapist adherence.

A CTAM score of 65 or above is considered to be indicative of sound methodological vigour and therefore good quality research with regard to the evaluation of psychological interventions in mental health (Tarrier and Wykes, 2004). Four of the 15 studies (26.67%) in this review scored above this cut off. Given that the review includes two articles employing single n methodology, two studies reporting on a case series and five un-controlled studies the quality scores were predicted to be lower due to the nature of research designs employed.

### Findings: PTS and trauma related outcomes

#### CBT

Four case studies/series reported on CBT delivered individually. The two case studies demonstrated reductions in PTS symptoms following treatment. One case no longer met criteria for PTSD and saw simultaneous reductions in trauma cognitions relative to pre-treatment scores (Kevan et al., 2007) and for the other case PTS scores fell below clinical range (Kayrouz and Vrklevski, 2015). Both cases in Callcott et al. (2004) study demonstrated reductions in symptoms, with one case moving to below clinical range. The same pattern was found for the case series by Hamblen et al. (2004) where two cases no longer met diagnostic criteria for PTSD and gains were maintained at 3 month follow-up. Three additional studies reported on CBT delivered individually and all drew upon a protocol designed specifically for individuals with SMI (Rosenberg et al., 2004; Mueser et al., 2008; Lu et al., 2009). All showed a significant effect of treatment on PTS symptoms.

In an un-controlled pilot study (Rosenberg et al., 2004) 11/12 participants showed a significant reduction in PTS symptoms. For the majority these gains were maintained or further reduced at 3 month follow-up. Significantly fewer participants met diagnostic criteria at follow-up (50%) compared to baseline (100%). Mueser et al. (2008) RCT also showed a significant reduction in symptoms for the CBT group compared to TAU. In their study, CBT proved to be no more effective than TAU in participants no longer meeting diagnostic criteria for a PTSD diagnosis. The study further showed that the effect size of change in PTS symptoms and meeting diagnostic criteria increased for participants with baseline PTS symptoms in the severe range and the effect size greatly reduced for those with a mild to moderate severity. This suggests a greater benefit of treatment for those with more severe symptoms. In keeping with these results, Lu et al. (2009) study conducted with an ethnically diverse sample (58% non-European American) showed significant reductions in PTS symptoms post-treatment and at 3 and 6 month follow-up. There was a significant reduction in the number of participants meeting diagnostic criteria for PTSD across all time points compared to baseline using the PCL. This was also the case for the PDS with comparisons between baseline and at all time points, excluding post-treatment.

Two studies reported on CBT delivered in a group format where treatment effects were demonstrated. Compared to a supportive counselling group, the CBT group saw significant reductions in PTS symptoms post-treatment (Trappler and Newville, 2007). Mueser et al.'s pilot study (Mueser et al., 2008) found significant reductions in PTS symptoms post-treatment and at 3 month follow-up. In this latter study, the number of people meeting PTSD diagnostic criteria was significantly reduced at all time points compared with baseline. On related PTS measures, the Mueser et al. study (Mueser et al., 2008) showed trauma cognitions were significantly reduced post-treatment and at follow-up compared with baseline. Knowledge of PTSD also increased post-treatment but the gains were not maintained at follow up. Frueh et al. study (Frueh et al., 2009) reported on an intervention integrating a mixed group/individually delivered protocol developed specifically for the treatment of PTSD in SMI groups. Results showed that PTS symptoms significantly reduced post-treatment and at 3 month follow-up compared to baseline. Ten of the 13 participants no longer met diagnostic criteria for PTSD at follow-up.

Jackson et al. (2009) study investigating the impact of CRI indicated that participants reported fewer PTS symptoms than the TAU group; with a borderline significant difference between the two groups. Post-treatment and at 12 month follow-up, significantly more participants in the CRI group showed a clinically significant change in PTS symptoms (≥25% reduction from baseline score) than the TAU group. This finding had a small to modest effect size. Baseline level of PTS symptoms predicted post-treatment PTS score, where participants with a higher baseline PTS score benefited most from CRI. A baseline score above clinical cut off on the IES saw a mean reduction of 28 points in comparison to a mean reduction of six points for those scoring below clinical cut off at baseline. Duration of untreated psychosis was also associated with treatment response, where participants with a shorter DUP were seen to benefit most from CRI.

#### PE

One study compared PE with EMDR using a randomised sample of ten participants with five participants in each group (de Bont et al., 2013b). The pooled treatment results in both intention to treat and completer groups showed self-reported PTS symptoms significantly reduced from pre-treatment through the treatment phase, post-treatment and then at follow-up with large effect size. The CAPS score was also shown to be significantly reduced at follow-up, although yielded borderline significant results at post-treatment. Of the four treatment completers in the PE condition, three no longer met diagnostic criteria for PTSD at post-treatment and this was increased to four participants at 3 month follow-up. When directly comparing PE with the EMDR condition, the findings suggested response to both treatments was comparable. Similarly, the RCT comparing EMDR, PE and waiting list TAU (van den Berg et al., 2015), reporting on a total sample of 155 participants showed that PE (*n* = 53) was associated with a significant reduction in PTS symptoms compared to waiting list control (*n* = 47) at post-treatment and at 6 month follow-up. Participants in the PE condition (and EMDR condition) were less likely to meet diagnostic criteria for PTSD post-treatment compared to those in waiting list control condition. Participants in the PE condition were also shown to be more likely to achieve full remission of PSTD symptoms compared with the wait-list control. PE was superior to EMDR in this respect.

#### EMDR

van den Berg and van der Gaag (2012) conducted a pilot investigation of EMDR, employing an un-controlled study design reporting on 27 participants. Following EMDR, 77.3% of the treatment completers no longer met diagnostic criteria for PTSD. The severity score reduced by 42.4 and 52.6% in intention to treat and completer groups, respectively. Significant improvements in self-reported PTS symptoms were found post-treatment compared to baseline. All significant findings had a large effect size. Similarly the RCT comparing EMDR, PE and waiting list control (van den Berg et al., 2015) showed that EMDR (*n* = 55), was associated with a significant reduction in PTS symptoms compared to waiting list control at post-treatment and at 6 month follow-up. Participants in the EMDR condition (and PE) were less likely to meet diagnostic criteria for PTSD post-treatment compared to those in waiting-list control condition. The study by de Bont et al. (2013b), with five participants randomised to the EMDR condition, as reported above, demonstrated a significant reduction in self-reported PTS symptoms at pre-treatment through the treatment phase, post-treatment and then at follow-up compared with pre-treatment scores all with a large effect size. Similar reductions in clinician rated PTS scores were too shown. Of the four treatment completers in the EMDR condition, three no longer met diagnostic criteria for PTSD at post-treatment and this was maintained at 3 month follow up.

#### Written emotional disclosure

One RCT (Bernard et al., 2006) investigated the use of written emotional disclosure. The study reported on 23 participants and found a significant reduction in severity of PTS symptoms for the intervention group between baseline and follow-up compared to the control group. Furthermore, significantly more participants in the intervention group (83.3%) reported a reduction in PTS severity compared to the control group (40%). The interaction between group and PTS severity accounted for 17% of the variance, indicating a small effect size. A main effect was found for avoidance symptoms, with lower avoidance ratings found at follow-up compared to baseline. This finding was only found for the intervention group, where a significant avoidance and group interaction was observed. There were no significant effects found for intrusion or arousal symptoms.

### Findings: outcomes in secondary domains

#### CBT

For the studies investigating CBT delivered in an individual format, the case studies/series demonstrated additional improvements in patient reports of self-trust and anxiety (Kayrouz and Vrklevski, 2015), reductions in symptoms of depression (Callcott et al., 2004; Kevan et al., 2007; Kayrouz and Vrklevski, 2015), general psychopathology (Callcott et al., 2004; Hamblen et al., 2004) and negative symptoms of psychosis (Callcott et al., 2004). In keeping with the findings outlined above, there too was a significant reduction in ratings of general psychopathology as measured by the BPRS found at 3 month follow-up but not immediately post-treatment in the pilot study conducted by Rosenberg et al. (2004). Specifically, the affect subscale on the BPRS significantly improved. The study by Lu et al. (2009) too showed significant improvements on the BPRS at 3 and 6 month follow-up, but not at post-treatment. Mueser et al. (2008) also found significant reductions of general psychopathology. Studies reported findings of significantly reduced depression (Mueser et al., 2008; Lu et al., 2009) and anxiety (Mueser et al., 2008). CBT was also associated with reduced health related concerns and improved ratings of the working alliance between the client and case manager (Mueser et al., 2008).

Group CBT appeared to have an impact on general psychopathology (Trappler and Newville, 2007) and on mood, where significant reductions on the BDI were observed post-treatment and at 3 month follow-up compared to baseline (Mueser et al., 2007). CBT delivered initially in a group then exposure delivered in an individual format demonstrated additional gains in ratings of anger and satisfaction post-treatment and at 3 month follow-up compared to baseline ratings (Frueh et al., 2009). General psychopathology also significantly improved at 3 month follow-up compared to baseline. This study found no effect of CBT on symptoms of depression, anxiety or social functioning/engagement. In keeping with these findings, the CRI study showed no significant differences for depression or self-esteem between the CRI treatment condition and TAU (Jackson et al., 2009).

#### PE

de Bont et al. (2013b) included a measure of general psychopathology and distress (OQ-45) and the pooled treatment results of both PE and EMDR showed a significant reduction in total scores from pre-treatment to post-treatment and follow-up. There was no effect found for social functioning as measured by the social functioning scale (SFS). The pooled results also showed no treatment effect on psychotic symptoms following treatment, however there was a significant reduction in psychosis-prone thinking on the O-life seen pre-treatment to post-treatment; which was not maintained at 3 month follow-up.

#### EMDR

The van den Berg and van der Gaag (2012) pilot study included secondary outcome measures of psychotic symptoms measured by the PSYRATS and symptoms of paranoia measured by the GPTS. A small significant reduction was found post-treatment on the PSYRATS subscales and total score however there was no significant difference found for paranoia. This is in contrast to the de Bont et al. (2013b) study which as outlined above found no treatment effect on the PSYRATS. A reduction post-treatment was found for psychosis-prone thinking which was not maintained at follow-up.

In the van den Berg et al. study (van den Berg and van der Gaag, 2012), post-treatment there were significant reductions in symptoms of depression and anxiety, and improvements in self esteem compared to baseline for both treatment completers and intention to treat groups. A significant difference was not found for ratings of hopelessness. As above, de Bont (de Bont et al., 2013b) showed a significant reduction in general psychopathology and distress from pre-treatment to post-treatment and follow-up. There was no effect found for social functioning.

#### Written emotional disclosure

Secondary outcome measures included measures of depression, anxiety, recovery style and insight. There were no significant findings with regard to the impact of the intervention on these domains. The results showed a main effect on insight with greater insight seen at follow-up compared with baseline however this did not differ significantly between groups and cannot therefore be interpreted as an effect of the intervention.

## Discussion

### Summary

#### What psychological interventions have published data?

The review identified 15 studies that had been published in peer reviewed journals reporting on psychological interventions targeting PTS symptoms in psychosis. The studies reported on individual and group interventions employing case studies, case series, un-controlled and controlled designs. More than two thirds of the studies included in the review reported on interventions which were cognitive behaviour therapy (CBT) informed. The review also described the findings of studies investigating eye-movement desensitisation and reprocessing (EMDR), prolonged exposure (PE) and written emotional disclosure.

#### How effective are the interventions in reducing PTS symptoms?

Overall, the studies indicated that psychological interventions are effective in reducing PTS symptoms, as they are shown to be in non-psychosis populations (Bisson and Andrew, 2005). Some studies however, demonstrated a delayed treatment effect with more significant reductions in PTS symptoms occurring following treatment. Some were not superior to TAU in reducing qualifying criteria for PTSD diagnosis, yet they were superior in reducing PTS symptoms. Many of the studies demonstrated maintenance of effect at follow-up comprising varying time points (e.g., 3 or 6 month follow-up). CBT protocols specifically designed for use in SMI populations, CBT used in ethnically diverse samples and CBT delivered in varying formats all demonstrated reduced PTS symptoms post-treatment. Cognitive recovery intervention (CRI) also showed a benefit in reducing PTS symptoms.

It seems important to highlight the heterogeneity of the CBT interventions included in the review. Five studies used a common protocol developed specifically for PTS symptoms within SMI; however the remaining six studies all varied in the degree to which any one aspect of a CBT intervention was prioritised, focused upon or not employed during sessions, with idiosyncratic adaptations for the use in an SMI sample. Although limited in the number of studies reporting on interventions other than CBT, the results for EMDR and PE were shown to be comparably effective in reducing PTS symptoms and associated cognitions. Written emotional disclosure too offered positive findings in terms of reducing PTS symptoms.

Psycho-education, relaxation training, exposure (Frueh et al., 2009) and written trauma elaboration (Kevan et al., 2007) were the treatment components associated with significant changes in PTS symptoms in these studies. Further, PTS symptoms were shown to be mediated by trauma related beliefs (Mueser et al., 2008). This is consistent with studies of trauma focused cognitive behavioural therapy (TF-CBT) in non-psychosis populations, which highlights the modification of trauma-related beliefs as a key mechanism of change (Kleim et al., 2013). Taken together, it would suggest the active treatment components and processes thought to bring about change in “general” PTSD samples might apply to those with a primary diagnosis of psychosis.

#### How effective are the interventions on secondary domains?

For CBT, EMDR, and PE, therapeutic benefits were seen consistently across studies with regard to measures of general psychopathology. Low mood and anxiety was improved following EMDR and PE, however there were mixed findings for CBT. The interventions also had inconsistent effects on psychotic symptoms. EMDR had a positive effect on self-esteem, however CBT did not. CBT was also associated with improvements in self-trust, health concerns, anger, ratings of working alliance and satisfaction. No intervention had an effect on social functioning. Written emotional disclosure was not associated with any secondary improvements in other domains.

### Additional considerations

The studies highlight a number of interesting findings and areas for consideration. Firstly, several studies found CBT to be more effective in reducing PTS symptoms for participants with higher pre-treatment PTS scores; suggesting those with more severe PTS symptoms will benefit most from CBT. Interestingly this is in contrast to the PTSD literature in the general population which shows that high pre-treatment PTS symptoms predicts high PTS symptoms post-treatment (Blanchard et al., 2003).

Homework was also implicated in the effectiveness of the intervention as greater homework completion contributed to improved outcomes for PTS symptoms and other secondary domains including depression and anxiety (Mueser et al., 2008). This is a consistent finding within the psychotherapy literature which sees the completion or engagement in homework tasks having a small to moderate effect size in predicting treatment outcome in CBT trials (Mausbach et al., 2010).

Psychological treatments, like other aspects of health care, need to be efficacious in the treatment of the targeted area of distress/difficulty and also accessible, appropriate and acceptable to the client group (Tarrier et al., 2006). Many of the studies reported on the occurrence of adverse events (e.g., significant increase in symptoms, hospitalisation and suicidality) and numbers of participants withdrawing from the study. A meta-analysis showed the average drop-out rate for trauma focused interventions is 18% (Imel et al., 2013). The drop-out rates of the CBT, PE, and EMDR studies (not including case studies/series) ranged from 14 to 41%. All of the studies with lower retention rates were investigating CBT. It is unclear if CBT is therefore a therapy this population is less able to engage in as there is a lack of data on alternative interventions and therefore direct comparison is currently limited. Written emotional disclosure yielded lower attrition rates with no drop outs in the experimental group. The results suggested that only one participant was lost at follow-up in the control group due to reasons unrelated to the trial. Although only a single study, and therefore with limited generalisability, it raises questions as to the value of non-therapist led interventions. A recent meta-analysis for example has shown a small to moderate effect size for “self help” interventions within psychosis highlighting this as an area for further utilisation and investigation (Scott et al., 2015).

Overall, the treatments presented in this review were reported to be safe to use in a SMI population. It was acknowledged, however, that trauma focused interventions, irrespective of whether the individual has a co-morbid psychosis diagnosis or not, can lead to distress (Devilly and Spence, 1999), and initial worsening of PTSD symptoms, emotional exhaustion and other physical symptoms of anxiety during the exposure phase of the treatment (Shearing et al., 2011; Hundt et al., 2016). No adverse events were reported in the majority of the studies. In a small proportion of cases where symptom exacerbation was reported, the participant either reported it was caused by factors unrelated to the intervention or was associated with the exposure aspects of treatment. In one CBT study however, exposure was not related to any exacerbation of symptoms and there were no drop outs at this stage. Participants expressed high treatment satisfaction and credibility. The majority of drop outs here occurred in the stabilisation phase. There was mixed implementation across the studies as to the use of a stabilisation or preparatory phase prior to beginning the trauma focused components of the intervention. A recent review suggests that there is insufficient evidence to support a phased based treatment approach to complex PTSD, i.e., specifically regarding a stabilisation phase, and that the inclusion of such a phase may in itself act as a delay or barrier to intervention targeting the trauma (De Jongh et al., 2016). It is worth noting however that as psychotic symptom exacerbation was only measured after the first two exposure sessions (van den Berg et al., 2015) further research is required to build on this evidence base regarding the safety and efficacy of treating post-traumatic stress in psychosis.

### Placing the findings in the current context

There are a number of findings within the literature that should be noted when considering the evidence relating to trauma-focused psychological interventions in psychosis. One finding is simply the utility and clinical benefit of being able to talk about the difficult experiences one has had. A study investigating the relationship between PTS symptoms arising as a result of first episode psychosis and self-disclosure found fewer PTS symptoms related to increased levels of disclosure about the traumatic experiences (Pietruch and Jobson, 2012). The study highlighted that disclosing and therefore talking about the experience would be beneficial and support recovery. This provides a clinical rationale that health professionals can communicate to individuals when broaching the topic of trauma assessment and treatment. This finding is important in that it provides evidence to counter the belief clinicians often hold that asking clients about traumatic experiences will in some way “make things worse” (Frueh et al., 2006). These types of beliefs have been identified as a common and notable barrier to the development and evaluation of psychological interventions targeting PTS symptoms in SMI groups (Salyers et al., 2004).

Related to encouraging open dialogue about traumatic experiences, there is evidence for the benefit of purely psycho-educational interventions for PTSD. Pratt et al. (2005) provided a three session PTSD psycho-education programme to individuals with primary SMI diagnoses whom all had co-morbid PTSD. The study did not aim to target PTS symptoms; outcomes showed increased knowledge of PTSD and high levels of satisfaction. Importantly, the authors reported many of the participants expressed an increased wish to access an intervention to help with these experiences. Considering that within clinical services, a proportion of service users are often reluctant to engage in psychological therapies (Berry and Haddock, 2008), this finding offers implications for normalisation, education and improving the degree to which interventions are deemed accessible. It must be noted however that this was not formally assessed or followed up in this study. These two findings taken together highlight the potential benefit of greater communication about PTS symptoms and the implications this has not only for engagement in therapies but also directly on recovery and outcome.

### Strengths and limitations

A main strength of the current review is the use of broad search criteria and the inclusion of published articles employing a range of study designs. The use, for example, of a broad trauma search strategy reduced the chance of potentially relevant records not being identified at this stage. Within the current psychosis literature, trauma exposure and symptoms arising as a direct result of these experiences are described heterogeneously. Terms such as trauma, traumatic event and adverse experiences are frequently all used to describe the same concept. Similarly PTSD, PTS symptoms, PTS response and traumatic reaction are also often used interchangeably. Despite the inherent issues arising as a result of a lack of consensus regarding terminology; the review's search methodology was nonetheless reflective of the language within the literature and thus thorough in its efforts to provide an accurate inclusive representation of the current research. The review is inclusive of all relevant findings within the literature independent of study design. In an area of emerging evidence, the exclusion of findings from studies employing less rigorous designs may not provide a comprehensive representation of the available research. The use of the CTAM (Tarrier and Wykes, 2004) has allowed for a transparent account of the evidence base clearly noting the heterogeneity of methodological vigor of the studies presented.

The review however is not without its limitations. The main limitation is, perhaps, simultaneously, also its main strength; the inclusion of un-controlled studies. The use of a controlled study design, in particular RCT, is considered the gold standard of investigating the true efficacy and effectiveness of an intervention (Jones and Podolsky, 2015). On the CTAM, the uncontrolled studies had lower quality ratings than controlled studies. There is evidence to suggest studies with poorer methodology overestimate the magnitude of positive benefits of the intervention; where a significant negative correlation between effect size and CTAM score has been found (Tarrier and Wykes, 2004). It must be noted that the CTAM was not designed to be used to evaluate case studies and therefore it may not have been a tool that accurately measured the quality of this type of study methodology. The lower scores therefore may not necessarily reflect the true quality of this type of research. Case studies by their nature are based on the work completed with people found within routine clinical services, and are therefore representative of the clients the practitioners accessing the research will be exposed to.

Another limitation of the review is the mixed population reported in several studies. The use of mixed samples therefore makes it difficult to infer the effectiveness of these interventions for the psychosis population specifically. Data were rarely stratified by diagnosis and therefore impossible to draw out these particular findings within this review. However, when considering the results of the nine pure psychosis studies, it is encouraging that outcomes suggested a beneficial impact of treatment on symptoms of PTS which provides promising support for use of these interventions in this group.

### Clinical and research implications

Firstly, the studies included within the review all report on the prevalence of high levels of trauma exposure within individuals with psychosis; which is in keeping with the literature (Bechdolf et al., 2010). The importance of routine trauma assessment and investigation across the illness of the negative sequelae is indicated (Read and Ross, 2003). This idea is consistent with a “screen and treat” methodology employed following the London terrorist bombings in 2005. Using this outreach approach, Brewin et al. (2008) identified significant numbers of people with a traumatic response to the bombings (and offered treatment) in comparison to the very small numbers of people who were referred independently of the screening process. This is of importance especially in the psychosis and SMI population where help seeking can be poor and complicated by other issues such as stigma and fear about the subsequent consequences (Kessler et al., 2001). Engagement and access is an ongoing area that needs to be addressed especially in light of evidence from this review that these individuals can nonetheless engage and benefit from trauma focused interventions such as TF-CBT and EMDR. This is in keeping with the NICE 2005 clinical guidance for the management of PTSD in non-psychosis groups, which recommends these two interventions as the first line treatment options. The review therefore appears to provide support for the applicability of the current PTSD NICE guidance to individuals with co-morbid severe mental health problems.

With NICE guidance for the care of people with experience of psychosis calling for routine screening as standard practice, solutions to overcome the additional impact of barriers at a staff and organisation level must too be sought. A recent study showed that a tailored training programme was successful in increasing staff confidence and knowledge in assessing and treating trauma within psychosis (Berry and Haddock, 2008). It is recommended that all teams employ a proactive informed approach to trauma because although emerging, there is sound evidence for the benefits of psychological interventions within this group.

This review demonstrated the relatively small number of RCTs that have been conducted in the area. Further methodologically vigorous controlled trials investigating the use of psychological interventions are required to be able to draw more solid conclusions about therapy efficacy; mirroring the call for an “*adequately powered, multi-centre RCT*” of a “*CBT based trauma reprocessing intervention*” in the recent NICE guidance. There also remains a need for research that extends targeting PTS symptoms alone. The review shows that the impact of the interventions on symptoms of psychosis is mixed; arguably as these were not the focus of the treatment. It may be interesting however to explore whether current trauma focused approaches can be adapted to optimise treatment gains by integrating components of psychosis focused therapies. Furthermore, new research should be extended beyond CBT as more studies looking at alternative interventions are indicated.

Interestingly, no studies were able to demonstrate functional gains such as change in employment status or number of health care visits. There was also a lack of participant reported outcomes such as measures of quality of life. A focus on both of these domains would be a welcome addition to further research in measuring the subjective impact of the interventions and evaluation of areas of importance for service users. This approach would be consistent with recovery focused services increasingly employed in psychosis (Warner, 2009). It may also provide a more meaningful evidence base that can support therapy engagement.

Trauma arising as a result of psychosis symptoms and hospital experiences is a topic that has received increasing recognition and interest more recently, as it has been shown that a significant number of individuals are meeting criteria for PTSD following these experiences (Berry et al., 2013). In support of this, Bernard and colleagues (Bernard et al., 2006) reported that the majority of their sample in the emotional written disclosure condition wrote about the “*debilitating and threatening effect of positive symptoms* (e.g., voices, delusions, hallucinations and paranoia)” and “*negative experiences such as being sectioned*.” Research with a focus on the development of PTS reactions to psychosis and hospitalisation may help to extend the current understanding about the impact and suitable interventions. Furthermore, studies evaluating interventions specifically targeting these post-psychotic PTS symptoms would be warranted.

## Conclusions

Traumatic experiences are implicated in the development of psychosis conditions and high rates of historical trauma are found in this group. People with psychosis are at higher risk of further trauma exposure. These individuals have greater service use and achieve poorer clinical outcomes including an increased risk of suicidality. There is a fast developing evidence base investigating trauma in psychosis with over 500 articles having been published in the last 5 years. This review presents the current evidence for the use of psychological interventions targeting PTS symptoms in a psychosis population. The review provided encouraging support for the efficacy of CBT, EMDR, PE, and written emotional disclosure suggesting the current NICE guidelines for the management of PTSD are clinically relevant to groups with co-morbid severe mental health difficulties such as psychosis. Although the evidence base has grown, there is a need for further research with a focus on mechanisms of change, patient reported outcomes and trials, particularly other than CBT, and employing rigorous research design within ethnically diverse more representative samples.

## Author contributions

SS, NK, NR and JO contributed to the conception, analysis, interpretation, drafting, critical revision and final approval of the manuscript for publication.

## Funding

A small amount of funding was provided from the Doctorate in Clinical Psychology Training Programme at the Institute of Psychiatry, Psychology and Neuroscience, King's College London. Open access for this article was funded by King's College London.

### Conflict of interest statement

The authors declare that the research was conducted in the absence of any commercial or financial relationships that could be construed as a potential conflict of interest.
