# Supplementary material for: Psychological Interventions for Post-traumatic Stress Symptoms in Psychosis: A Systematic Review of Outcomes
Source: Front Psychol. 2017 Mar 14;8:341. doi: 10.3389/fpsyg.2017.00341 (PMC5348513; doi:10.3389/fpsyg.2017.00341)
Supplement: Supplementary file 1 [file Table1.pdf]

Supplementary Material

Psychological interventions for post-traumatic stress symptoms in psychosis: A systematic review of outcomes

Sarah Swan[1*], Nadine Keen[1,2], Nicola Reynolds[1,2], Juliana Onwumere[1,2]

[1] South London and Maudsley NHS Foundation Trust, London, United Kingdom
[2] Department of Psychology, Institute of Psychiatry, Psychology and Neuroscience, King's College London, United Kingdom

***Correspondence**
Dr Sarah Swan
Sarah.Swan@slam.nhs.uk

1 Supplementary Tables

1.1 Supplementary Table 1. Characteristics of studies investigating psychological interventions for PTS symptoms in the context of a primary diagnosis of psychosis

Authors (Date)	Country	Design	Sample Size N (Psychosis %)	Setting & Format	Intervention	No. of sessions	Outcome measures	Key results
Callcott, Standart & Turkington (2004)	UK	Case series	2 (100%)	Community<br><br>Individual	CBT	12 to 17	IES, BDI, CPRS, SANS	Reduction in PTS symptoms in both cases, including intrusions; one case showed reduced avoidance strategies and depressive symptoms; other case improved on general psychopathology and negative symptoms of psychosis.
Hamblen, Jankowski, Rosenberg & Mueser (2004)	USA	Case series	3 (66.6%)	Community<br><br>Individual	CBT	12 to 16	CAPS, BPRS	Following intervention, two cases no longer meeting criteria for PTSD; reductions in general psychopathology.<br><br>At 3 month follow-up, end of therapy gains were maintained.<br><br>One case still meeting criteria for PTSD showed clinically meaningful reduction in PTS symptoms and general psychopathology.
Rosenberg et al (2004)	USA	Un-controlled study	22 (45%)	Community & inpatient<br><br>Individual	CBT	12 to 16	CAPS, BPRS, Safety measures	Statistically and clinically significant reduction in PTS symptoms following intervention<br><br>At 3 month follow-up, reduction in PTS symptoms maintained; significantly fewer participants meeting criteria for PTSD compared to baseline; significant reduction in affect symptoms.

Bernard, Jackson & Jones (2006)	UK	RCT	23 (100%)	Community<br><br>Individual	Written Disclosure vs Writing of non-emotional topics	3	IES-R, RSQ, IS, HADS, PANAS	Significant reduction in severity of PTS symptoms for treatment group compared to control.<br><br>There was a main effect for avoidance, with reduced avoidance symptoms seen at follow-up compared to baseline, when comparing treatment with control.
Kevan, Gumley & Coletta (2007)	UK	Case study	1 (100%)	Community<br><br>Individual	CBT	9	PDS, PTCI, BDI-II,	No longer meeting diagnostic criteria for PTSD following treatment; reduction in negative cognitions and self-blame; clinically significant reduction in depression (severe to minimal range); general effect of treatment in reducing subjective ratings of problematic beliefs.<br><br>Gains maintained at 1 month follow-up.
Mueser et al (2007)	USA	Un-controlled study	80 (21%)	Community<br><br>Group	CBT	21	THQ, PCL, K-PTSD, PTCI, BDI-II	Significant reduction in PTS symptoms and number of participants meeting diagnostic criteria; significant reduction in trauma related cognitions; knowledge about PTSD significantly increased; significant reduction in depression; treatment completers had fewer PTS symptoms and depression at the end of the treatment compared to non-completers.<br><br>At 3 month follow-up, gains maintained expect knowledge about PTSD.

Trappler & Newville (2007)	USA	Non random-ised controlled study	48 (100%)	Inpatient<br><br>Group	CBT vs Supportive counselling group	12	IES, BPRS	Significant reduction in PTS symptoms, specifically regarding intrusions and avoidance, for the CBT group compared to supportive counselling; Significant decrease in positive symptoms, emotional withdrawal, tension, depression, unusual thought content, blunted affect, excitement, disorientation and elevated mood for CBT group.
Mueser et al (2008)	USA	RCT	108 (15.7%)	Community<br><br>Individual	CBT vs TAU	12 to 16	CAPS, PTCI, K-PTSD, BPRS, BAI, BDI-II, SF-12, WAI	CBT significantly better in reducing PTS symptoms; no difference between CBT and TAU in eliminating PTSD diagnosis however; larger effect size seen for more severe PTS symptoms compared to mild-moderate symptom severity; CBT was more effective in reducing negative trauma-related cognitions, depression, anxiety, health related concerns and improving knowledge of PTSD.<br><br>At 3 and 6 month follow-up, gains continued to improve.
Frueh et al (2009)	USA	Un-controlled study	20 (100%)	Community<br><br>Mixed: Group followed by individual sessions	CBT	22	CAPS, PCL, HAM-A, CGI, NAI, MOS SF-36, CPOSS, Function-ing	Significant reduction in PTS symptoms at end of treatment; significant improvement in symptoms of anger, satisfaction, quality of social relationships.<br><br>At 3 month follow-up, PTS symptoms significantly improved (>75% treatment responders); anger and satisfaction significantly improved compared to baseline; fewer primary care visits endorsed compared to baseline.

Jackson et al (2009)	UK	RCT	66 (100%)	Community<br><br>Individual	CBT (CRI) vs TAU	up to 26	IES-R, CDSS, SCQ	Borderline significant difference between CRI and TAU for PTS symptoms; higher baseline severity of PTS symptoms and shorter duration of untreated psychosis tended to benefit the most from CRI; no difference between groups for symptoms of depression or self esteem.<br><br>At 12 months follow-up, significantly more participants in the CRI group made clinically significant improvements compared to TAU.
Lu et al (2009)	USA	Un-controlled study	19 (16%)	Community<br><br>Individual	CBT	12-16wks	PDS, PCL, BPRS, BDI-II	Over 75% of participants had a significant improvement in PTS symptoms; significantly fewer participants meeting criteria post-treatment.<br><br>Significantly fewer participants meeting PTSD criteria compared to baseline at 3 and 6 month follow-up; 92% and 100% of participants showed significant reduction in PTS symptoms compared to baseline at 3 and 6 month follow-up respectively.
Van den Berg & Van der Gaag (2012)	Netherla nds	Un-controlled study	27 (100%)	Community<br><br>Individual	EMDR	6	CAPS, PSS-SR, PSYRATS (AHRS & DRS), GPTS, BDI-II, BAI,BHS, SERS-SF	Significant reduction in PTS symptoms (large effect size); small but significant reduction in psychotic symptoms in treatment completers; depression, anxiety and self esteem significantly improved.

De Bont, Van Minnen & De Jongh (2013)	Netherlands	RCT	10 (100%)	Community<br><br>Individual	PE vs EMDR	12	CAPS, PSS-SR, PSYRATS (AHRS), O-LIFE, OQ-45, SFS, safety measures	PTS symptom severity significantly reduced; 75% of completers no longer met criteria for PTSD; significant reduction in general psychopathology; no significant effect on psychotic symptoms however significant decline in psychosis-prone thinking. Outcome was comparable between PE and EMDR with no significant difference.<br><br>At 3 month follow-up, gains in PTS symptoms maintained.
Kayrouz & Vrklevski (2015)	Australia	Case study	1 (100%)	Inpatient<br><br>Individual	CBT	19	IES-R, DASS,TABS, qualitative measure of psychosis	At post intervention a 70% reduction in PTS symptom report; no longer in clinical range for mood/anxiety symptoms; increase in social engagement.<br><br>Elevation in paranoia during treatment which recovered post-treatment.
Van den Berg et al (2015)	Netherlands	RCT	155 (93.6%)	Community<br><br>Individual	PE, EMDR vs Waiting List (TAU)	8	CAPS, PSS-SR, PTCI	PE and EMDR reduced PTS symptoms/cognitions and were more likely to achieve and maintain loss of PTSD diagnosis compared to TAU; there was no significant difference in outcome when comparing PE and EMDR.<br><br>At 6 month follow-up, outcome was maintained for both PE and EMDR

BAI = Beck anxiety inventory; BDI=Beck depression inventory; BHS=Beck hopelessness scale; BPRS=Brief psychiatric rating scale; CAPS=Clinician administered PTSD scale; CGI=Clinical global impressions scale; CPOSS=Charleston psychiatric outpatient satisfaction scale; CPRS=Comprehensive psychopathological rating scale; CDSS=Calgary depression scale; DASS=Depression anxiety stress scale; GPTS=Green et al paranoid thought scale; HADS=Hospital anxiety and depression scale; HAM-A= Hamilton rating scale for depression; IES/IES-R=Impact of events scale/revised; IS=Insight scale; K-PTSD=Knowledge of PTSD test; MOS-SF36/SF-12=Medical outcome study short form health survey; NAI=Novaco anger inventory; O-LIFE=Oxford liverpool inventory of feelings and experiences; OQ-45=Outcome questionnaire; PANAS=Positive and negative affect schedule; PCL=PTSD checklist, PSS-SR=PTSD symptom scale self report; PSYRATS=Psychotic symptom rating scale (AHRS=auditory hallucination rating subscale, DRS=Delusion rating subscale); PTCI=Post-traumatic cognitions inventory; RSQ=Recovery style questionnaire; SANS=Scale for assessment of negative symptoms; SCQ=Self esteem questionnaire; SERS-SF=Self esteem rating scale short form; SFS=Social functioning scale; TABS=Trauma and attachment beliefs scale; THQ=Trauma history questionnaire; WAI=Working alliance inventory

frontiers
